# Factors associated with patient activation among patients with diabetes on hemodialysis: a multicenter cross-sectional study from a developing country

**DOI:** 10.1186/s12882-024-03674-z

**Published:** 2024-07-20

**Authors:** Jehad M. Zeidalkilani, Yazan A. Milhem, Reem N. Shorafa, Sari Taha, Amer A. Koni, Samah W. Al-Jabi, Sa’ed H. Zyoud

**Affiliations:** 1 Department of Medicine, College of Medicine and Health Sciences, An-Najah National University, Nablus, 44839 Palestine; 2 An-Najah Global Health Institute (GHI), An-Najah National University, Nablus, 44839 Palestine; 3 Department of Public Health, Faculty of Medicine and Health Sciences, An-Najah National University, P.O. Box 7, Nablus, Palestine; 4 Department of Anatomy, Biochemistry and Genetics, An-Najah National University, Nablus, 44839 Palestine; 5 Department of Clinical and Community Pharmacy, College of Medicine and Health Sciences, An-Najah National University, Nablus, 44839 Palestine; 6 Division of Clinical Pharmacy, Department of Hematology and Oncology, An-Najah National University Hospital, Nablus, 44839 Palestine; 7 Poison Control and Drug Information Center (PCDIC), College of Medicine and Health Sciences, An-Najah National University, Nablus, 44839 Palestine; 8 Clinical Research Centre, An-Najah National University Hospital, Nablus, 44839 Palestine

**Keywords:** Patient activation, Diabetes mellitus, Chronic kidney disease, Hemodialysis, Health-related quality of life, HRQOL

## Abstract

**Background:**

Diabetes mellitus (DM) is a major public health concern with considerable morbidity and mortality. DM affects patients’ quality of life and can lead to multiple complications, including chronic kidney disease (CKD) and the need for dialysis. Higher patient activation can improve health outcomes in hemodialysis patients with DM. This study aimed to explore the factors associated with higher patient activation and health-related quality of life (HRQoL) among hemodialysis patients with DM.

**Methods:**

This was a cross-sectional, questionnaire-based study conducted on hemodialysis patients with DM in Palestine. The quota sampling method was utilized to draw samples from six dialysis centers. The questionnaire consists of three sections. The first section includes demographic, socioeconomic and clinical questions. The second section utilizes the patient activation measure-13 (PAM-13) to measure patient activation, while the third section assesses HRQoL using the EQ-5D-5 L tool and the visual analog scale (VAS). Mann‒Whitney and Kruskal‒Wallis tests were employed to examine the relationships between variables at the bivariate level, and multiple regression analysis was employed at the multivariate level.

**Results:**

Of the 200 patients who were approached, 158 were included. The median PAM, EQ-5D index, and VAS score were low at 51.0, 0.58, and 60.0, respectively. A higher PAM score was independently associated with a higher household income level and taking medications independently. A higher EQ-5D index was associated with taking more than eight medications, taking medications independently, living with fewer than three comorbid conditions, and having a higher PAM. A higher VAS score was associated with being married, and receiving less than 3.5 hours of hemodialysis.

**Conclusions:**

A higher patient activation level was associated with a higher income level and independence in taking medications. Interventions designed to improve patient activation, such as medication management programs, should address these factors among the target population. Longitudinal studies are needed to assess the time effect and direction of causation between health status and patient activation.

## Background

Diabetes mellitus (DM) is a leading global health problem associated with substantial morbidity and mortality. The global prevalence of DM doubled from 1990 to 2019, directly contributing to 1.5 million deaths [1]. DM affects the quality of life of patients due to its chronic nature and the need for continuous self-management, including blood glucose monitoring, following a certain diet, engaging in regular exercise and adhering to medication regimens [2]. Moreover, DM can lead to various complications, such as diabetic nephropathy and retinopathy, resulting in physical, functional and social challenges [3]. Of note, DM is among the most common causes of chronic kidney disease (CKD) [4]. The prevalence of CKD in patients with type 2 DM ranges from 23.1 to 41.7% [5–7]. Progression to dialysis is 2.7 times more common in patients with DM than in those without DM [8]. Furthermore, once dialysis is initiated, patients with DM have a higher mortality rate than those without DM [9].

The progression of DM to CKD is governed by several factors, such as tight glycemic control, systolic blood pressure, age, albuminuria and duration of DM [10]. In particular, intensive glycemic control has a well-established role in reducing the risk of progression to hemodialysis in patients with DM [11]. However, the recommended level of glycemic control for patients with DM on dialysis is different and variable, and evidence on reducing morbidity and mortality is scarce [12–14].

Patient activation is the readiness and ability of patients to manage their own health and well-being and adopt certain health behaviors to improve their conditions. To do so, patients need the motivation, knowledge, skills, and confidence necessary to make decisions and manage their own care [15, 16]. High activation in patients with DM was found to be associated with fewer admissions, better glycemic control, blood glucose monitoring, physical exercise, improved nutritional habits, and adherence to recommended eye examinations and foot care [17–19]. Likewise, patient activation was associated with increased home-based dialysis and better blood pressure control in hemodialysis patients [20, 21]. However, patients with CKD demonstrated less patient activation than patients with other chronic diseases, with even lower activation levels in those receiving inpatient dialysis [22, 23]. One study revealed a low level of patient activation among patients with DM on dialysis, which was associated with older age and poorer self-reported health [24].

Between 2006 and 2021, the number of patients receiving hemodialysis in the Palestinian West Bank almost quadrupled, peaking at 1554 patients [25, 26]. These patients were found to have a high prevalence of malnutrition and poor quality of life [27, 28]. In 2015, the average annual cost per patient was estimated at 16,085 USD, inclusive of medications, tests and outpatient visits [29]. This high cost is covered by the governmental healthcare system, which is underfunded, fragmented, and aid dependent [30]. As engaged patients demonstrate increased adherence to treatment and follow-up and improved self-care, patient activation reduces costs by decreasing costly service utilization, such as hospital admissions and emergency department visits [16, 31]. Therefore, patient activation provides an opportunity to improve health outcomes and save costs, especially for patients with conditions requiring considerable self-management, such as DM and the need for hemodialysis. Identifying the extent and drivers of patient activation among this population is key to informing interventions and designing guidelines aimed at enhancing patient activation. This study aimed to measure the level of patient activation among hemodialysis patients with DM and explore the factors associated with patient activation. The study also aimed to assess the health-related quality of life (HRQoL) of this population.

## Methods

### **Study design and settings**

This was a multicenter, cross-sectional study based on a self-administered questionnaire to measure patient activation, health-related quality of life (HRQoL) and associated factors among patients who were diagnosed with DM and receiving hemodialysis. The study was conducted in six dialysis centers in the northern West Bank in the cities of Nablus, Tulkarem, Jenin, Qalqilya, Tubas and Salfit.

### Population and sampling

The study population comprises patients with DM currently receiving hemodialysis in the northern Palestinian West Bank. According to the annual health records published by the Palestinian Ministry of Health, 507 patients received hemodialysis in the northern governorates of the Palestinian West Bank. As almost 45% of those on hemodialysis are estimated to have DM, the estimated total population size is 230 patients [32]. To estimate the sample size, the Raosoft online sample size calculator was used (http://www.raosoft.com/samplesize.html). Using a margin of error of 5% and a confidence level of 99%, the sample size was calculated to be 171. The target sample size was increased to 200 to account for potential missing data. The respondents were chosen using the quota sampling method corresponding to the distribution of the population in the 6 centers.

### Inclusion and exclusion criteria

Patients who (1) were over 18 years of age, (2) had been diagnosed with DM for more than one year prior to the study, or (3) had been receiving hemodialysis regularly for at least six months before the study were included. Patients with cognitive or mental limitations were excluded.

### Data collection: procedures and tools

Permission was obtained to access medical records at each dialysis center to identify patients with DM undergoing hemodialysis who could participate in the study as per the inclusion and exclusion criteria. Participants, including those who could not read, were invited to participate, interviewed face-to-face, and assisted in filling out the questionnaire.

The questionnaire was developed in Arabic, and the tools were properly translated. A pilot study was carried out on a sample of 20 respondents, based on which the questionnaire was edited for ease, clarity and accuracy. The questionnaire is structured into three sections:

- The first section included questions on demographic characteristics, including age, sex, height, weight; socioeconomic characteristics, including residency, marital status, education level, occupation, household income; and clinical characteristics, including dialysis vintage (< 4 or ≥ 4 years), duration of DM (≤ 10, 11–20, or > 20 years), frequency of dialysis (< 3 or ≥ 3 sessions/week), duration of each session (< 3.5 or ≥ 3.5 h/session), history of kidney transplantation (yes/no), number of chronic comorbid diseases (< 3 or ≥ 3), number of chronic medications (< 8 or ≥ 8 medications/day), and ability to take medications independently (yes/no). The variable categorization was based on previous similar studies [32–34].
- The second section assessed patient activation using the Patient Activation Measure-13 short form (PAM-13), which is a 13-item questionnaire that measures the patient’s knowledge, skills, and confidence necessary for self-management of health and health conditions. Responses are based on a 4-point Likert scale (from strongly disagree to strongly agree). The final score is calculated based on these responses and ranges from 0 to 100. Then, the final score is used to assign respondents to one of four levels of patient activation, with levels 1 and 2 indicating lower patient activation and levels 3 and 4 indicating higher patient activation [35]. The PAM-13 is among the most widely translated and validated tools for measuring patient activation and has been tested for validity and reliability in different locations and for various populations [35–38], including patients with CKD [39].
- The third section assesses HRQoL using the EuroQol tool (EQ-5D-5 L). This tool is easy and practical for clinical use and has been validated in patients with CKD before and after receiving hemodialysis [40]. It consists of two parts. The first part is the five-dimensional, five-level EuroQol tool (EQ-5D-5 L). This part measures health status in five dimensions (mobility, self-care, usual activities, pain and discomfort, and anxiety and depression), with each domain assessed at five levels of response (no problems, slight problems, moderate problems, severe problems, and extreme problems). Then, the health state index score is calculated using a formula that assigns an index value to each level of each dimension. The index value is different for different regions to reflect the societal perspective of different health states. The final health state index score ranges from 0 (health status of dead) to 1 (best health possible). The second part is the visual analog score (VAS), whereby respondents estimate their perceived health from 0 (the worst health status) to 100 (the best health status) [41].

### Data analysis

Descriptive and inferential statistics were employed to analyze the data using the 26th version of the Statistical Package for the Social Sciences (SPSS). Cronbach’s alpha was used to test the internal consistency between the PAM and EQ-5D scale items, with a value greater than 0.70 indicating an acceptable level of internal consistency as a type of scale reliability [42]. Percentages and frequencies are reported for the categorical and ordinal independent variables of sociodemographic and clinical characteristics. The frequency, percentage, median, mean rank and/or mean (± standard deviation) were reported for the scale scores (PAM, VAS and EQ-5D).

The inferential statistical tests chosen to analyze the data were based on the median and mean rank as measures of central tendency, as parametric data assumptions, such as normality of the data and equality of variance, are not met. The values of the scale scores (PAM, VAS and EQ-5D) can be skewed for several reasons. First, the population consists of individuals with chronic conditions who, for instance, may demonstrate a low level of activation, leading to a left-skewed distribution [43]. The perception of health can be influenced by other population characteristics, such as cultural differences and social expectations of better health status. Furthermore, scales often have a limited range restricted by upper and lower limits, and thus, reporting of the highest and lowest possible scores is not uncommon, leading to ceiling and floor effects [44]. Additionally, as the exact numerical differences between the score values as ordinal data may not be meaningful, the data cannot be normally distributed; thus, the median is a more representative measure of central tendency [45, 46]. Therefore, the Mann‒Whitney test was used when comparing two groups, while the Kruskal‒Wallis test was used for more than two groups. Furthermore, to ensure the nonnormality of the data, the Shapiro‒Wilk test was used to test for normality. The mean rank, as the average based on ranking all observations, is used to calculate the *H*-value that is necessary for nonparametric tests, such as the Kruskal‒Wallis test [47]. The multivariate analysis employed a multiple linear regression model that included all the factors that demonstrated significant associations with the PAM, EQ-5D, and VAS scores at the bivariate level. The significance level was set at *p* < 0.05.

## Results

**Fig. 1:**
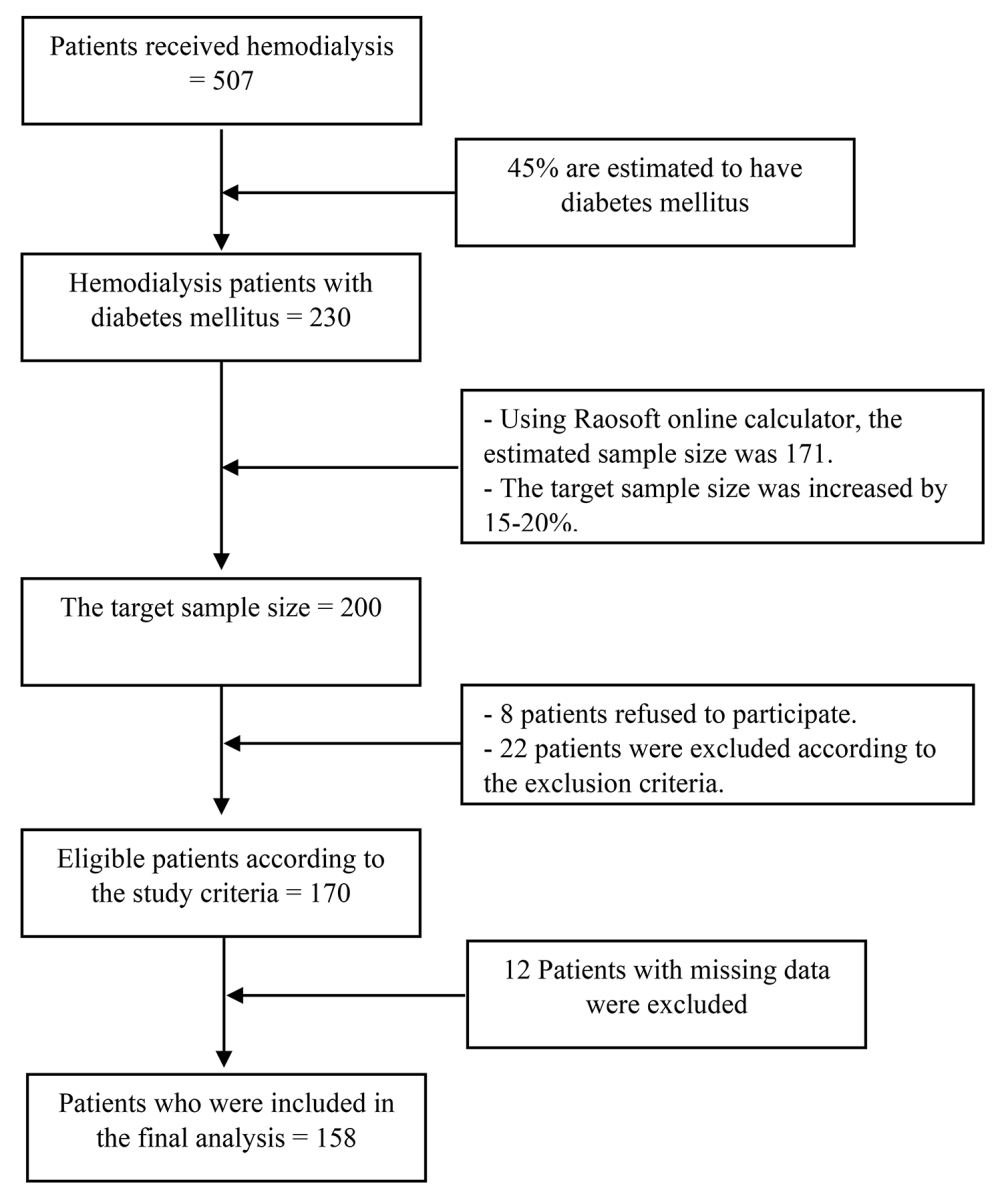
CONSORT diagram of the included hemodialysis patients

Of the 200 patients with DM who were approached, eight declined to participate (4%), 22 were excluded based on the exclusion criteria (11%), and 12 were excluded because of considerable missing data (Fig. 1). Among the final sample of 158 patients, 99 (62.7%) were males, and 59 (37.3%) were females. The mean age of the participants was 60.92 years (*SD* ± 10.65), and almost half of the participants were younger than 60 years. The vast majority of participants were unemployed (93.7%), and almost two-thirds had a low monthly income.

Only 29 patients (18.4%) had been diagnosed with DM for less than 10 years, while 57 patients (36.1%) had been diagnosed for more than 20 years. Most patients reported taking fewer than eight medications a day (62.7%) and taking their medications independently (60.1%). The majority had been on hemodialysis for less than 4 years (68.9%), had undergone hemodialysis more than 3 times a week (90.5%), and had undergone hemodialysis for more than 3.5 h per session (64.6%) (Table 1).

**Table 1:** Sociodemographic and clinical characteristics of the samples in this study

Variable	Frequency (%) *N* (158)
**Age category**	
< 60 ≥ 60	78 (49.4) 80 (50.6)
**Sex**	
Male Female	99 (62.7) 59 (37.3)
**BMI** ^a^	
Underweight or Normal Overweight Obese	55 (34.8) 53 (33.6) 50 (31.6)
**Education**	
No formal education Primary Secondary High School University	15 (9.5) 35 (22.2) 35 (22.2) 30 (19) 43 (27.2)
**Household income (month)**	
< 2000 NIS 2000–5000 > 5000	103 (66.2) 48 (30.4) 7 (4.4)
**Residency**	
City Village Palestinian refugee camps	45 (28.5) 103 (65.2) 10 (6.3)
**Current Living**	
Alone With family	7 (4.4) 151 (95.6)
**Marital status**	
Single, divorced, widowed Married	20 (12.7) 138 (87.3)
**Occupation**	
Unemployed Employed	148 (93.7) 10 (6.3)
**Current Smoking status**	
Non-smoker Smoker	38 (24.1) 120 (75.9)
**Dialysis vintage (Years)**	
< 4 ≥4	109 (69) 49 (31)
**Diabetes (Years)**	
≤10 11–20 >20	29 (18.4) 72 (45.6) 57 (36.1)
**Dialysis per week**	
<3 ≥3	15 (9.5) 143 (90.5)
**Dialysis session duration (hours)**	
<3.5 ≥3.5	56 (35.4) 102 (64.6)
**Transplantation history**	
Yes No	2 (1.3) 156 (98.7)
**Total chronic comorbid diseases**	
<3 ≥ 3	111 (70.3) 47 (29.7)
**Chronic medications (per day)**	
< 8 ≥ 8	99 (62.7) 59 (37.3)
**Taking medications alone**	
Yes No	95 (60.1) 63 (39.9)
**Patient Activation Measure**	
Not believing activation important A lack of knowledge and confidence to take action Beginning to take action Taking action	37 (23.4) 59 (37.3) 47 (29.7) 15 (9.6)
**Visual analog score**	
0–20% 21–40% 41–60% 61–80% 81–100%	5 (3.2) 30 (19.0) 66 (41.8) 50 (31.6) 7 (4.4)

Abbreviations: BMI = body mass index

The PAM, EQ-5D, and VAS scores were found to have a nonnormal distribution (*p* < 0.001). The Cronbach’s alpha values of the PAM and EQ-5D were 0.72 and 0.80, respectively, indicating an acceptable level of internal consistency. The median PAM score was 51.0 (IQR = 48.9–58.1). At the bivariate level, a higher household income level (*p* = 0.002), the use of more than eight medications (*p* = 0.037) and the ability to take medications independently (*p* = 0.026) were significantly associated with PAM. No significant associations were found with age, sex, education, employment status or marital status. At the multivariate level, a higher household income level (*p* < 0.001, β = 0.271) and ability to take medications independently (*p* = 0.001, β = 0.257) were significantly related to the PAM score, while the number of medications taken by the patient was not significantly different (*p* = 0.557, β = 0.588). The mean rank was highest for those with the highest household income (137.07) and lowest for those with no formal education (63.13) (Table 2).

**Table 2:** Patient activation scores and sociodemographic and clinical variables among diabetic patients on hemodialysis (*n* = 158)

Variable	Frequency (%) *N* = 158	Median [1st Q-3rd Q]	*P* value * (bivariate analysis)	Multivariate analysis		Mean Rank
				*P* value*	Regression coefficient	
**Age category**						
< 60 ≥ 60	78 (49.4) 80 (50.6)	51[51-58.10] 51[47-58.10]	0.659 ^a^	**-**	**-**	81.11 77.93
**Sex**						
Male Female	99 (63.7) 59 (37.3)	51[48.9–58.10] 51[48.9–58.10]	0.873 ^a^	-	**-**	79.94 78.75
**BMI** ^a^						
Underweight or Normal Overweight Obese	55 (34.8) 3 (33.6) 50 (31.6)	51[48.9–60.60] 51[47-58.10] 53[51.2-58.73]	0.552 ^b^	-	**-**	78.10 75.70 85.07
**Education**						
No formal education Primary Secondary High School University	15 (9.5) 35 (22.2) 35 (22.2) 30 (19) 43 (27.2)	51[42.2–60.60] 51[43.7–55.60] 53.2[41.6–58.10] 53.2[45.47–60.60] 51[47-60.60]	0.221 ^b^	-	**-**	63.13 69.71 80.61 88.63 85.90
**Household income (month)**						
< 2000 NIS 2000–5000 > 5000	103 (66.2) 48 (30.4) 7 (4.4)	51[48.90–58.10] 51[47-60.60] 65.50[60.60–75]	**0.002** ^b^	**< 0.001**	0.271	75.18 80.38 137.07
**Residency**						
City Village Refugee camps	45 (28.5) 103 (65.2) 10 (6.3)	51[51-59.35] 51[48.90–58.10] 49[46.58–60.53]	0.708 ^b^	-	-	78.09 80.83 68.50
**Current living**						
Alone With family	7 (4.4) 151 (95.6)	51[42.2–60.60] 51[43.7–55.60]	0.362 ^a^	-	-	64.29 80.21
**Marital status**						
Single, divorced, widowed Married	20 (12.7) 138 (87.3)	51[45.53–55.60] 51[48.90–60.60]	0.202 ^a^	-	-	67.45 81.25
**Occupation**						
Unemployed Employed	148 (93.7) 10 (6.3)	51[48.90–58.10] 51[47.60-66.68]	0.862 ^a^	-	-	79.34 81.90
**Current smoking status**						
Non-smoker Smoker	38 (24.1) 120 (75.9)	51[51-60.60] 51[47-58.10]	0.461 ^a^	-	-	84.21 78.01
**Dialysis vintage (years)**						
< 4 ≥4	109 (69) 49 (31)	51[48.90–58.10] 51[47-58.10]	0.716 ^a^	-	-	80.38 77.55
**Diabetes duration (years)**						
≤10 11–20 >20	29 (18.4) 72 (45.6) 57 (36.1)	51[46.15–56.85] 51[48.90–60.60] 51[51-59.35]	0.317 ^b^	-	-	68.21 81.03 83.31
**Dialysis per week**						
<3 ≥3	15 (9.5) 143 (90.5)	51[47-65.50] 51[48.90–58.10]	0.952 ^a^	-	-	80.17 79.43
**Dialysis session duration (hours)**						
<3.5 ≥3.5	56 (35.4) 102 (64.6)	51[47-63.65] 51[50.48–58.10]	0.949 ^a^	-	-	79.19 79.67
**Transplantation history**						
Yes No	2 (1.3) 156 (98.7)	61.8 [58.1–65.5] 51 [48.9–58.1]	0.143 ^a^	-	-	126 78.90
**Total chronic comorbid diseases**						
<3 ≥ 3	111 (70.3) 47 (29.7)	51[51-58.10] 51[45.30–58.10]	0.231 ^a^	-	-	82.30 72.88
**Chronic medications (per day)**						
< 8 ≥ 8	99 (62.7) 59 (37.3)	51[47-58.10] 55[51-60.60]	**0.037** ^a^	0.557	0.588	73.68 89.26
**Taking medications alone**						
Yes No	95 (60.1) 63 (39.9)	53.20[51-60.60] 51[45.30–55.60]	**0.026** ^a^	**0.001**	0.257	73.32 89.86

Abbreviations: BMI = body mass index

* Bold values denote statistical significance at the *p* < 0.05 level

^a^ Statistical significance was measured using the Mann‒Whitney U test

^b^ Statistical significance was measured using the Kruskal‒Wallis test

The median EQ-5D index was 0.58 (IQR = 0.32–0.80). At the bivariate level, age less than 60 years (*p* = 0.047), male sex (*p* = 0.014), higher educational level (*p* < 0.001), household income (*p* = 0.040), being married (*p* = 0.007), living with fewer than three comorbid conditions (*p* = 0.030), taking eight medications or more (*p* = 0.003), taking medications alone (*p* = 0.023), and having a PAM (*p* = 0.002) were significantly associated with a higher EQ-5D index. At the multivariate level, the EQ-5D index retained significant associations with living with fewer than three comorbid conditions (*p* = 0.041, β =-0.160), taking more than eight medications (*p* = 0.003, β = 0.231), taking medications alone (*p* = 0.048, β = 0.154), and having a higher PAM level (*p* = 0.012, β = 0.190) while age (*p* = 0.195, β =-0.092), sex (*p* = 0.0339, β =-0.073), educational level (*p* = 0.159, β = 0.120), household income level (*p* = 0.647, β = 0.036), and marital status (*p* = 0.066, β = 0.134) showed no significant associations. The mean rank was highest for those with a history of kidney transplantation (131.00) and lowest for those with no formal education (42.17). Only two patients had a history of kidney transplantation (Table 3). The frequencies and proportions of patients’ responses across the domains of the EQ-5D tool are depicted in Table 4.

**Table 3:** Total EQ-5D score and sociodemographic and clinical variables among diabetic patients receiving hemodialysis (*n* = 158)

Variable	Frequency (%) *N* = 158	Median [1st Q-3rd Q]	*P* value * (bivariate analysis)	Multivariate analysis		Mean Rank
				*P* value*	Regression coefficient	
**Age category**						
< 60 ≥ 60	78 (49.4) 80 (50.6)	0.60[0.34–0.88] 0.55[0.20–0.72]	**0.047** ^**a**^	0.195	-0.092	86.8 72.38
**Sex**						
Male Female	99 (63.7) 59 (37.3)	0.62[0.35–0.87] 0.51[0.19–0.70]	**0.014** ^**a**^	0.339	-0.073	86.37 67.97
**BMI** ^a^						
Underweight or Normal Overweight Obese	55 (34.8) 53 (33.6) 50 (31.6)	0.53[0.23–0.76] 0.61[0.47–0.82] 0.53[0.17–0.80]	0.335 ^b^	-	-	75.86 87.07 75.48
**Education**						
No formal education Primary Secondary High School University	15 (9.5) 35 (22.2) 35 (22.2) 30 (19) 43 (27.2)	0.18[-0.02-0.56] 0.55[0.21–0.66] 0.53[0.40–0.79] 0.63[0.21–0.77] 0.72[0.51–0.88]	**0.001** ^**b**^	0.159	0.120	42.17 71.26 82.67 77.83 97.81
**Household income (month)**						
< 2000 NIS 2000–5000 > 5000	103 (66.2) 48 (30.4) 7 (4.4)	0.53[0.33–0.72] 0.61[0.26–0.87] 0.87[0.76-1.00]	**0.040** ^**b**^	0.647	0.036	74.69 84.30 117.29
**Residency**						
City Village Refugee camps	45 (28.5) 103 (65.2) 10 (6.3)	0.58[0.31–0.79] 0.58[0.29–0.77] 0.60[0.38–0.91]	0.820 ^b^	-	-	79.11 78.82 88.30
**Current Living**						
Alone With family	7 (4.4) 151 (95.6)	0.63[0.33–0.72] 0.58[0.29–0.79]	0.836 ^a^	-	-	76 79.66
**Marital status**						
Single, divorced, widowed Married	20 (12.7) 138 (87.3)	0.36[0.00-0.63] 0.59[0.34–0.84]	**0.007** ^**a**^	0.066	0.134	53.93 83.21
**Occupation**						
Unemployed Employed	148 (93.7) 10 (6.3)	0.56[0.30–0.77] 0.68[0.51-1.00]	0.129 ^a^	-	-	78.06 100.75
**Current Smoking status**						
Non-smoker Smoker	38 (24.1) 120 (75.9)	0.63[0.29–0.92] 0.55[0.32–0.76]	0.243 ^a^	-	-	87.04 77.11
**Dialysis vintage (Years)**						
< 4 ≥4	109 (69) 49 (31)	0.60[0.32–0.84] 0.53[0.20–0.71]	0.083 ^a^	-	-	83.72 70.11
**Diabetes duration (Years)**						
≤10 11–20 >20	29 (18.4) 72 (45.6) 57 (36.1)	0.67[0.09–0.87] 0.56[0.26–0.77] 0.60[0.39–0.76]	0.848 ^b^	-	-	78.95 75.67 84.62
**Dialysis per week**						
<3 ≥3	15 (9.5) 143 (90.5)	0.72[0.53-1.00] 0.56[0.28–0.76]	0.066 ^a^	-	-	100.17 77.33
**Dialysis session duration**						
**(hours)** <3.5 ≥3.5	56 (35.4) 102 (64.6)	0.67[0.34–0.88] 0.55[0.29–0.75]	0.077 ^a^	-	-	88.19 74.73
**Transplantation history**						
Yes No	2 (1.3) 156 (98.7)	0.88[0.76-1.00] 0.58[0.30–0.79]	0.109 ^a^	-	-	131 78.84
**Total chronic comorbid diseases**						
<3 ≥ 3	111 (70.3) 47 (29.7)	0.60[0.38–0.84] 0.47[0.16–0.69]	**0.030** ^**a**^	**0.041**	-0.160	84.63 67.39
**Chronic medications (per day)**						
< 8 ≥ 8	99 (62.7) 59 (37.3)	0.51[0.18–0.76] 0.66[0.52–0.84]	**0.003** ^**a**^	**0.003**	0.231	71.04 93.70
**Taking medications alone**						
Yes No	95 (60.1) 63 (39.9)	0.63[0.43–0.87] 0.40[0.10–0.71]	**0.023** ^**a**^	**0.048**	0.154	89.97 63.71
**Patient Activation Measure**						
• Not believing activation important • A lack of knowledge and confidence to take action • Beginning to take action • Taking action	37 (23.4) 59 (37.3) 47 (29.7) 15 (9.6)	0.34[0.09–0.57] 0.58[0.34–0.76] 0.63[0.35–0.84] 0.87[0.56-1.00]	**0.002** ^**b**^	**0.012**	0.190	54.09 81.22 87.62 109.97

Abbreviations: BMI = body mass index

* Bold values denote statistical significance at the *p* < 0.05 level

^a^ Statistical significance was measured using the Mann‒Whitney U test

^b^ Statistical significance was measured using the Kruskal‒Wallis test

**Table 4:** Frequencies and proportions of patients’ responses across the domains of the EQ-5D tool (*n* = 158)

	Mobility Frequency (%)	Self-care Frequency (%)	Usual activities Frequency (%)	Pain/discomfort Frequency (%)	Anxiety/depression Frequency (%)
**Level 1 – no problem**	52 (32.9)	77 (48.7)	57 (36.1)	60 (38.0)	62 (39.2)
**Level 2 – Slight problems**	31 (19.6)	27 (17.1)	20 (12.7)	46 (29.1)	52 (32.9)
**Level 3 – Moderate problems**	30 (19.0)	17 (10.8)	31 (19.6)	38 (24.1)	22 (13.9)
**Level 4 – Severe problems**	31 (19.6)	13 (8.2)	15 (9.5)	14 (8.9)	18 (11.4)
**Level 5 – Extreme problems/unable to do**	14 (8.9)	24 (15.2)	35 (22.2)	0 (0.0)	4 (2.5)
**Total**	158 (100)	158 (100)	158 (100)	158 (100)	158 (100)

The median VAS score was 60.0 (IQR = 45.0–70.0). At the bivariate level, a higher household income level (*p* = 0.012), being married (*p* = 0.013), a hemodialysis session duration of 3.5 h or less (*p* = 0.019), taking eight chronic medications or more (*p* = 0.037), and a higher PAM level (*p* = 0.015) were significantly associated with a higher VAS score. At the multivariate level, being married (*p* = 0.020, β = 0.180), and having a hemodialysis session duration of less than 3.5 h (*p* = 0.018, β =-0.185) were significantly related to the VAS score, while household income level (*p* = 0.274, β = 0.087), the PAM level (*p* = 0.323, β = 0.078), and taking eight or more chronic medications (*p* = 0.059, β = 0.151) were not significantly related to the VAS score. The mean rank was highest for those with the highest household income (128.86) and lowest for those who were not married (55.85) (Table 5).

**Table 5:** Visual analog scale scores and sociodemographic and clinical variables among diabetic hemodialysis patients (*n* = 158)

Variable	Frequency (%) *N* = 158	Median [1st Q -3rd Q]	*P* value* (bivariate analysis)	Multivariate analysis		Mean Rank
				*P* value*	Regression coefficient	
**Age category**						
< 60 ≥ 60	78 (49.4) 80 (50.6)	60 [50–70] 60 [41.25-70]	0.807 ^a^	-	-	78.61 80.37
**Sex**						
Male Female	99 (63.7) 59 (37.3)	60 [45–70] 60 [50–70]	0.754 ^a^	-	-	78.63 80.97
**BMI** ^a^						
Underweight or Normal Overweight Obese	55 (34.8) 53 (33.6) 50 (31.6)	60 [40–70] 60 [50–65] 60 [45–70]	0.894 ^b^	-	-	81.79 78.68 77.85
**Education**						
No formal education Primary Secondary High School University	15 (9.5) 35 (22.2) 35 (22.2) 30 (19) 43 (27.2)	60 [40–70] 60 [50–65] 60 [50–65] 50 [40-66.25] 65 [45–70]	0.472 ^b^	-	-	77.83 78.27 78.74 69.05 88.99
**Household income (month)**						
< 2000 NIS 2000–5000 > 5000	103 (66.2) 48 (30.4) 7 (4.4)	60 [45–70] 60 [41.25-70] 70 [70–90]	**0.012** ^**b**^	0.274	0.087	76.26 79.25 128.86
**Residency**						
City Village Refugee camp	45 (28.5) 103 (65.2) 10 (6.3)	60 [40-67.50] 60 [50–70] 55 [43.75-70]	0.799 ^b^	-	-	76.11 81.26 76.60
**Current Living**						
Alone With family	7 (4.4) 151 (95.6)	60 [50–70] 60 [45–70]	0.915 ^a^	-	-	81.29 79.42
**Marital status**						
Single, divorced, widowed Married	20 (12.7) 138 (87.3)	47.5 [36.25-60] 60 [50–70]	**0.013** ^**a**^	**0.020**	0.180	55.85 82.93
**Occupation**						
Unemployed Employed	148 (93.7) 10 (6.3)	60 [45–70] 60 [35-62.5]	0.475 ^a^	-	-	80.17 69.60
**Current Smoking status**						
Non smoker Smoker	38 (24.1) 120 (75.9)	60 [40–65] 60 [45–70]	0.680 ^a^	-	-	76.86 80.34
**Dialysis vintage (Years)**						
< 4 ≥ 4	109 (69) 49 (31)	60 [45–70] 60 [50–70]	0.063 ^a^	-	-	79.08 80.43
**Diabetes duration (Years)**						
≤10 11–20 >20	29 (18.4) 72 (45.6) 57 (36.1)	60 [50–65] 60 [41.25-70] 60 [47.50–70]	0.843 ^b^	-	-	79.52 77.42 82.11
**Dialysis per week**						
<3 ≥3	15 (9.5) 143 (90.5)	60 [45–70] 60 [45–70]	0.947 ^a^	-	-	78.77 79.58
**Dialysis session duration**						
**(hours)** <3.5 ≥3.5	56 (35.4) 102 (64.6)	60 [50–70] 57.50 [45–65]	**0.019** ^**a**^	**0.018**	-0.185	90.94 73.22
**Transplantation history**						
Yes No	2 (1.3) 156 (98.7)	70 [60–90] 60 [40–70]	0.278 ^a^	-	-	116.75 79.02
**Total chronic comorbid**						
**diseases** <3 ≥ 3	111 (70.3) 47 (29.7)	60 [45–70] 60 [45–70]	0.456 ^a^	-	-	81.25 75.37
**Chronic medications (per day)**						
< 8 ≥ 8	99 (62.7) 59 (37.3)	60 [40–70] 60 [50–70]	**0.037** ^**a**^	0.059	0.151	73.68 89.26
**Taking medications alone**						
Yes No	95 (60.1) 63 (39.9)	60 [45–70] 60 [45–65]	0.285 ^a^	-	-	82.64 74.77
**Patient Activation Measure**						
• Not believing activation important • A lack of knowledge and confidence to take action • Beginning to take action • Taking action	37 (23.4) 59 (37.3) 47 (29.7) 15 (9.6)	60 [50–70] 55 [40–65] 60 [50–70] 70 [50–90]	**0.015** ^**b**^	0.323	0.078	84.18 68.26 80.44 109.23

Abbreviations: BMI = body mass index

* Bold values denote statistical significance at the *p* < 0.05 level

^a^ Statistical significance was measured using the Mann‒Whitney U test

^b^ Statistical significance was measured using the Kruskal‒Wallis test

## Discussion

This was the first study to measure patient activation in Palestine. In particular, this study aimed to assess patient activation and HRQoL among hemodialysis patients with DM. To date, only one study has measured patient activation among those with DM on dialysis globally [24]. The study findings revealed a low level of patient activation and HRQoL. A higher household income level and taking medications independently were associated with a higher PAM score, while other demographic constructs, such as educational level, employment and age, showed no significant association.

In this study, the median PAM was found to be low at 51.0. This is in line with similar studies conducted among patients on hemodialysis that reported measures ranging between 51 and 66.7 [23, 48–50]. In addition, other studies that were conducted among patients on dialysis indicated low activation, but comparisons to these studies are limited due to differences in methodology [24, 51]. Of note, these findings align with the prevailing pattern of lower patient activation in those with CKD than in those with other chronic conditions [22], which is even more pronounced in patients on hemodialysis than in those on peritoneal dialysis [52]. The complexity and chronicity of the specialized process of dialysis give rise to a unique culture where healthcare professionals are entirely responsible for service delivery, limiting patient engagement [53]. Moreover, dialysis imposes a substantial burden on patients’ physical and mental well-being due to the chronicity of the disease, presence of associated chronic conditions, and impact on quality of life [54–57]. Indeed, HRQoL was low among participants in our study and even lower in other studies conducted among patients on hemodialysis in Palestine [32, 33].

However, patients undergoing dialysis can be actively involved in self-management of certain aspects of the dialysis process. Patients can be assigned dialysis-related tasks that range in complexity from simple clinical observation, such as weight measurement, to complex tasks, such as dialysis machine programming [58, 59]. Moreover, the use of patient-reported outcome measures, whereby patients actively measure symptom burden and quality of life, can enhance patient agency and improve clinician-patient communication [60, 61]. Currently, two trials are testing the impact of using patient-reported outcome measures in hemodialysis care in Australia and Canada [62, 63].

The findings of this study revealed a positive association between household income level and patient activation. However, no correlation was found between education level or employment status and patient activation. Similar income associations were reported in most studies addressing patient activation generally and among those with DM [16, 18, 64–66]. In contrast to our study, education level was found to be associated with patient activation among adult patients [64, 66], patients undergoing dialysis [48, 67], and patients with DM [68, 69], heart failure [70], pre-dialysis CKD [71], and other chronic conditions [72]. Notably, the subconstructs of household income, educational level, and employment status may confound the relationship of each with patient activation. A study conducted among diabetic patients on dialysis revealed that socioeconomic status, a construct of income, education, and employment, was not associated with patient activation [24]. Similarly, a national survey of American adults revealed that the income association narrowed after controlling for education, which suggests that the income effect might be influenced by other social factors [64]. This highlights the importance of utilizing a more rigorous methodology in assessing social and economic constructs in relation to patient activation, whereby the multifaceted interactions between income, education, and employment are analyzed separately and collectively. This is especially crucial for translating research into real-world interventions because such social factors interact to shape patient activation. In addition, the study revealed that taking medications independently was associated with a higher PAM score, which corresponds with the definition of the active patient as capable and motivated to manage their own conditions [15, 35].

This study revealed no significant association between age and patient activation, which contradicts previous studies conducted on a similar population of patients with DM on dialysis [24] and other populations of patients with DM [18, 67–69], CKD [71], hypertension [73], osteoarthritis [36], and adult patients in general [64]. However, other studies conducted in different research settings have reported no such associations [65, 70, 74]. The impact of age on patient activation may vary among populations with different demographic and clinical characteristics. Even within populations sharing similar clinical characteristics, patient activation may be influenced by disparities in social, cultural and health-related factors, which may confound the effect of age. For instance, the presence of cognitive and physical challenges, social support, and health literacy can encourage or discourage patients from being able to manage their own conditions [75–77]. The lower patient activation in the younger population could be ascribed to the emotional distress resulting from the context of unexpected need for dialysis at a young age, which affects the willingness to actively engage in self-care. Moreover, the demanding lifestyle and career expectations of early- and middle-aged adults make younger patients less likely to devote their time and effort to their dialysis self-care.

Due to this variation in the influence of demographic, socioeconomic and clinical factors across different populations and the interplay between these factors within the same population, it is crucial to interpret research findings with caution. Researchers and policymakers should navigate the nuances and complexities associated with these factors to enhance research methodology and build effective interventions. Such interventions are better informed by studies conducted on populations with similar characteristics. Therefore, the results of our research may inform interventions aimed at improving patient activation, especially among the target population, by focusing on the factors influencing active engagement. For example, developing a medication management program for patients receiving dialysis in Palestine can improve knowledge, empower decision-making and thus encourage patient activation. A medication management program may include medication reviews, medication reconciliation, self-monitoring for side effects and drug interactions, and educational programs tailored to the literacy level of each patient [78, 79].

The use of more than eight chronic medications was positively associated with HRQoL, as measured by the EQ-5D index and VAS score. Three studies examined the relationship between medication number and quality of life in Palestine. One study did not reveal a significant association among the same population of hemodialysis patients with DM [33]. Nonetheless, the other two studies, conducted among patients undergoing hemodialysis regardless of diabetic status, found that taking fewer medications was associated with better quality of life [32]. The impact of the number of medications on quality of life depends on the type and appropriateness of the medications. Personalized, effective medications can improve symptoms, control chronic conditions, enhance functionality, and improve psychological well-being. However, drawing conclusions solely based on the number of medications can be misleading, as the choice and number of medications should be tailored to individual needs.

Moreover, several other factors were also associated with each of the two HRQoL scores used in this study. Living with fewer than three chronic conditions, taking medications independently, and having a higher PAM were associated with a higher EQ-5D score, and marital status and a duration of hemodialysis less than 3.5 h were associated with a higher VAS score. The marital status association is consistent with a study conducted among the same patient population in Palestine [33], in addition to other local [80], regional [81] and global studies [82–84] conducted on patients with DM and CKD. The association between marital status and HRQoL can be explained by the social support provided by married partners, which can improve the self-care and psychological well-being of patients [85]. Notably, research indicates that marital quality can be more important than marital status, especially in patients with DM [86]. Furthermore, other studies conducted on patients with DM, similar to the present study, reported a positive association between self-reported health, a component of HRQoL, and the PAM, which adds to the evidence on the benefits of patient activation on improving health outcomes [67, 87, 88]. However, the direction of causation remains uncertain. It is unclear whether a higher patient activation level may lead to better HRQoL or whether patients with better HRQoL may be more motivated to manage their own conditions. As this relationship may be bidirectional and operate in a virtuous cycle, longitudinal studies exploring differences across time may be needed to better elucidate the effect.

### Strengths and limitations

This was the first study exploring patient activation among hemodialysis patients with DM in Palestine, which can inform interventions and guide future research on patient activation. However, this study has a few limitations. First, the generalizability of the study findings is limited due to the potential influence of cultural, social, and clinical characteristics on patient activation. Second, the use of a subjective, self-reported scale might have introduced measurement errors resulting from personal perceptions, biases or social desirability. Third, some strata of the categorical variables, such as household income level and transplant history, had low frequencies, which might have affected the quality of analysis and the validity of the results. Finally, the cross-sectional design inherently restricts the ability to establish cause-and-effect relationships, thus limiting the applicability and understanding of how certain constructs, such as perceived health, affect patient activation.

## Conclusions

Higher patient activation can improve health outcomes and reduce healthcare-associated costs in hemodialysis patients with DM. This study explored patient activation, HRQoL, and the factors influencing both among hemodialysis patients with DM in Palestine. These patients demonstrated low levels of patient activation and HRQoL. A higher household income level and independence in taking medications were associated with a higher PAM score. Interventions targeting health literacy and improving capability in regard to medications, such as medication management programs, have the potential to improve patient activation among the target population. Future research should employ rigorous methodologies to investigate the complex and confounding relationships between factors influencing patient activation. In addition, longitudinal studies are needed to examine the temporal effect and the presence and direction of causation, particularly between HRQoL and patient activation.

## Data Availability

Due to privacy concerns, the datasets used and/or analyzed during the current study are available from the corresponding author upon reasonable request. This manuscript is part of a Doctor of Medicine graduation project submitted to An-Najah National University. The abstract was published as part of self-archiving institutional repositories (university repository: https://repository.najah.edu/bitstreams/5760440f-efcc-4650-a3c4-e34b18bb2300/download).

## Abbreviations

DM: diabetes mellitus
CKD: chronic kidney disease
HRQo: health-related quality of life
ESRD: End-stage renal disease
PAM-13: Patient activation measure-13
EQ-5D-5L: the five-dimensional, five-level EuroQol tool
VAS: visual analog scale
SBSS: Statistical Package for the Social Sciences
IQR: Interquartile Range
